# Surveillance of *Schistosoma japonicum* Infection in Domestic Ruminants in the Dongting Lake Region, Hunan Province, China

**DOI:** 10.1371/journal.pone.0031876

**Published:** 2012-02-16

**Authors:** Jinming Liu, Chunxia Zhu, Yaojun Shi, Hao Li, Lanpin Wang, Shangtian Qin, Saie Kang, Yanpin Huang, Yamei Jin, Jiaojiao Lin

**Affiliations:** 1 National Laboratory of Animal Schistosomiasis Control/Key Laboratory of Animal Parasitology, Ministry of Agriculture, Shanghai Veterinary Research Institute, Chinese Academy of Agricultural Sciences, Shanghai, People's Republic of China; 2 Hunan Animal Disease Control Center, Changsha, People's Republic of China; 3 Yueyang County Animal Husbandry Bureau, Yueyang, People's Republic of China; 4 Yuanjiang Municipal Animal Husbandry and Fishery Bureau, Yuanjiang, People's Republic of China; Pennsylvania State University College of Medicine, United States of America

## Abstract

**Background:**

Schistosomiasis japonica is prevalent in Asian countries and it remains a major public health problem in China. The major endemic foci are the marsh and lake regions of southern China, particularly the Dongting Lake region bordering Hunan and Hubei provinces, and the Poyang Lake region in Jiangxi province. Domestic ruminants, especially bovines, have long been considered to play a major role in the transmission of *Schistosoma japonicum* to humans.

**Methods and Findings:**

A miracidial hatching technique was used to investigate the prevalence of *S. japonicum* infections in domestic ruminants and field feces collected from two towns located to the south and east of Dongting Lake, Hunan province, between 2005 and 2010. The overall prevalence of infection was not significantly reduced from 4.93% in 2005 to 3.64% in 2008, after which it was maintained at this level. Bovines comprised 23.5–58.2% of the total infected ruminants, while goats comprised 41.8–76.5%. Infection rates in cattle and goats were significantly higher than those found in buffalo in most study years. The prevalence in buffalo younger than three years was significantly higher than that in those aged over three years. All the positive field samples of feces were derived from bovines in Nandashan. In Matang Town, 61.22% of the positive field feces were from bovines, while the rest were from goats. The positive rates for field feces were approximately the same in April and November/October.

**Conclusions:**

The present study found that bovines and goats are major sources of *S. japonicum* infection in the Dongting lake region and there was age-related resistance in buffalo. Both bovines and goats should be treated equally when controlling *S. japonicum* infections in the Dongting lake region. It is essential to conduct an additional mass treatment in late March or early April, in addition to the original treatment scheme.

## Introduction

Schistosomiasis is one of the most prevalent parasitic infections worldwide. According to the World Health Organization [1], the schistosomiasis disease burden is equivalent to 1.7 to 4.5 million disability-adjusted life years (DALYs) [2] lost worldwide each year, which is among the highest of all neglected tropical diseases. The global prevalence is currently estimated to be 207 million cases, with another 779 million people at risk of infection in 76 countries and territories [3], [4]. Approximately 120 million people are symptomatic and 20 million have severe and debilitating disease [5].

Schistosomiasis japonica is prevalent in many Asian countries and it remains a major public health problem in China, with an estimated 65 million people at risk of infection and 0.36 million individuals infected in 2009 [6]. The major endemic foci are the marsh and lake regions of southern China where more than 82% of infected individuals live [7], particularly the Dongting Lake region bordering Hubei and Hunan provinces, and the Poyang Lake region in Jiangxi province.

Of the five major schistosome species infecting humans, *Schistosoma japonicum* is the only species where zoonotic transmission is important. Over 40 mammal species act as definitive hosts of *S. japonicum* and one-third play an important role in disease transmission in China [8]. The Chinese national schistosomiasis control program considers only cattle and water buffalo to be significant nonhuman contributors to schistosomiasis transmission, based on their size, life expectancy, and infection intensity [9]. Sheep and goats are likely to contribute only minimally to overall transmission, because they have low fecal output and they are only present on marshlands for limited periods before they are sold at an early age as a food source [10]. However, we consider that sheep and goats may be very important to transmission in some endemic areas where they are found in large numbers with a high level of infection and generally allowed to graze freely on grassy lake beach areas near the residential area and outside but near embankments.

In 2004, the State Council of China established two targets for the National Schistosomiasis Control Program. The goal was to reduce the prevalence of infection in both humans and domestic animals to <5% by 2008, and then to <1% by 2015 in all endemic counties. A comprehensive control strategy was implemented widely in China to reach these targets, which was based on blocking the transmission of *S. japonicum* from cattle/buffalos and humans to snails [11]. To support this control strategy and better understand the kinetics of schistosomiasis in domestic animals and the role of ruminants in schistosomiasis transmission, we investigated infections of bovines and goats in two large townships near Dongting Lake region. We also surveyed environmental contamination with schistosome eggs from different animals. It was found that bovines and goats were both important sources transmitting *S. japonicum* infection to *Oncomelania* snails in the Dongting Lake region.

## Materials and Methods

### Ethics Statement

The study protocol was approved by the Scientific Steering Committee of the Shanghai Veterinary Research Institute (permit number: 2004-15). All animal owners were informed about the purpose and procedures of the study before being asked for their consent to participate. Permits for the described field studies were obtained from Yueyang County Animal Husbandry Bureau and Yuanjiang Municipal Animal Husbandry and Fishery Bureau.

### Study area

The study was conducted in two towns. Nandashan town is located on the south of Dongting Lake, in Yuanjiang City, Hunan Province. The study area in Nandashan town included six villages with 20854 people. In 2010, the area was estimated as containing 678 bovines, 38000 swine, and 380 other domestic animals, such as cats and dogs. Matang Town is located on the east of Dongting Lake, in Yueyang County, Hunan Province. The study region covered an area of 19 villages with 224 household groups and a human population of 178998. In 2010, the area was estimated to contain 1032 bovines, 560 goats, 28,000 swine, and 350 other domestic animals, such as dogs. In both towns, the intermediate host snail, *Oncomelania hupensis*, is distributed mainly in the marshland lake areas outside embankments, including grassy lake beaches. Water buffalo, cattle, and goats are generally allowed to graze freely on marshlands, whereas pigs are penned, and dogs mainly live in residential areas. Ruminant herds were usually removed from areas outside embankments during the flooding season in May to September. Bovines remained on the grassy beaches for the rest of the year while goats were penned at night. Most of the inhabitants are farmers who mainly grow rice, cotton, and wheat. A low number of farmers keep livestock and they frequently enter marshland. Human schistosomiasis prevalence remained approximately 10% in Nandashan town and 5% in Matang Town, between 2000 and 2005. *S. japonicum*-positive animals receive chemotherapy treatment with praziquantel in May/June and an additional bovine mass treatment during September/October has been applied annually in the decades prior to 2005. From 2005 to 2007, bovines and goats received two mass treatments in May or June, and September). From 2008 to 2010, the treatment scheme was same as that applied before 2005, although goats also received an additional mass treatment. Over 90% of bovines and 20–30% of goats were tested each May or June. The dose of praziquantel for each animal was determined based on its weight (30 mg kg^−1^ for cattle, 25 mg kg^−1^ for buffaloes, and 20 mg kg^−1^ for goats, which were orally administered). Pregnant animals were not treated. A comprehensive control strategy was introduced for both town in 2004, which was based on interventions to reduce the rate of transmission of *S. japonicum* infection among cattle (and buffalo), humans and snails [11]. Snail control efforts were mainly undertaken in the area inside embankments through the use of chemical molluscicides or changing the mode of agricultural production (e.g. converting paddy fields infested with *Oncomelania* snails into glebes in which to plant fruit or vegetables, or constructing fish ponds in valleys infested with *Oncomelania* snails).

### Survey of *S. japonicum* infection in ruminants

Veterinary workers interviewed all farmers in the villages to obtain data on species, number, and age of domestic ruminants present.

The prevalence of *S. japonicum* infection was investigated in May or June (before treatment) each year between 2005 and 2010, using a miracidial hatching technique. Bovine investigation coverage was at least 90% throughout the study. We randomly selected 20–25% of goats for investigation. All farmers in the surveyed villages were informed and requested to keep their animals penned and secured in the morning of collections to facilitate stool sample collection from each animal. The majority of the fecal samples were directly collected around animals, but some samples were obtained via the rectum. The weight of fecal samples collected was 150 g for water buffalo and cattle, and 30 g for goats. Three consecutive hatching tests were carried out with all fecal samples, according to the National Standard Procedures (Ministry of Agriculture, 2002). Thus, fecal samples were divided equally into three subsamples, before being suspended in water and filtered through 260 mesh per inch sieves (nylon tissue bag). Filtrates were then transferred to a flask and incubated under strong artificial or natural illumination at room temperature (25–30°C). The presence of miracidia was assessed at 1, 3, and 5 h after incubation.

The number of infected animals was calculated for each town and for each species based on the total number and prevalence.

### Survey of field feces collected from marshland

We selected four marshlands (each marshland occupied a ground space of 100×100 m^2^) that were located outside the embankments of each town and which contained the freshwater snail *O. hupensis*. Two surveys were conducted every year, with one in April and another in October/November (50–60 days after the mass treatment). All fresh fecal samples were collected from the surveyed marshlands (one dropping represented a sample; 15–40 samples from each marshland per investigation) and classified based on their source. The weight of each sample was 80–100 g for bovine feces and 20–30 g for goat feces.

The prevalence of *S. japonicum* infection in feces was investigated using the miracidial hatching technique described above (only one hatching test per sample). We tested 50 g for bovine feces and 10 g for goat feces.

### Statistical analysis

Data were double-entered into Microsoft Excel 2003 and cross-checked. The Chi-squared test was used to compare the infected number in different years, and the prevalence of *S. japonicum* among different animals, years, and age groups. Significance levels of 0.05 and 0.01 were used for all tests.

## Results

### Overall prevalence and the number of infected domestic ruminants

A total of 10537 ruminant fecal samples were examined between 2005 and 2010, and a total of 436 (4.14%) samples were positive for *S. japonicum* in both towns. The prevalence of infection reduced non-significantly (*P* = 0.28) from 4.93% in 2005 to 3.64% in 2008 and then remained at this level (Table 1). There was a significant decline in the total number of domestic ruminants (χ^2^ = 76.15, *P*<0.01) and the number of infected ruminants also dropped significantly from 201 to 89 (χ^2^ = 87.65, *P*<0.01). The most significant decline of the infected number was in goats from 130 to 23 (χ^2^ = 147.79, *P*<0.01), whereas the reduction of bovines was not significant (*P* = 0.75).

**Table 1:** Overall prevalence and the number of infected domesticated ruminants.

Year	Total No.	No. examined	No. positive	Prevalence (%)	Infected No.	
					Bovine*	Goat
2005	2794	1867	92	4.93	71(40)	130
2006	2756	1952	87	4.46	65(37)	93
2007	2504	1645	70	4.26	64(29)	54
2008	2287	1511	55	3.64	55(31)	40
2009	2260	1786	64	3.58	58(32)	25
2010	2270	1776	68	3.83	66(32)	23

*The infected number of bovines was the sum of numbers of cattle and buffaloes, which were calculated with their total number (data not shown) and prevalence (in table 2 and 3) from each town. The numbers in brackets were from Matang Town.

All infected ruminants in Nandashan Town were bovines (cattle and buffalo). In Matang Town, only 23.5–58.2% of infected ruminants were buffalo, whereas the remaining 41.8–76.5% were goats. Overall the infected ruminants from Nandashan and Matang Town were comprised of 35.3–74.2% bovines and 25.8–64.7% goats.

### Prevalence of *S. japonicum* infection in cattle and buffalo from Nandashan Town

Approximately 80% of grazing ruminants in Nandashan Town were buffalo, while the remaining 20% were cattle. A total of 3556 fecal samples were examined, i.e., 745 from cattle and 2811 from buffalo. The prevalence of *S. japonicum* infection was 4.40–8.47% in cattle and 2.77–5.59% in buffalo (Table 2), and there was a nonsignificant difference between years. The prevalence in cattle was higher than that in buffalo in each study year, and there was a significant difference in 2005 (*P*<0.01) and 2008 (*P*<0.05). The overall positive rate of cattle fecal samples (6.67%) was significantly higher than that found in buffalo (3.91%) (χ^2^ = 10.73, *P*<0.01).

**Table 2:** Prevalence of *S. japonicum* infection in cattle and buffalo from Nandashan Town.

Year	Cattle			Buffalo		
	No. examined	No. positive	Prevalence (%)	No. examined	No. positive	Prevalence (%)
2005	200	15	7.50**	494	14	2.81**
2006	135	6	4.40	448	19	4.20
2007	182	13	7.14	340	19	5.59
2008	99	7	7.07*	470	13	2.77*
2009	70	4	5.71	511	20	3.9
2010	59	5	8.47	548	25	4.56

*P<0.05;

**P<0.01 comparison of prevalence between cattle and buffalo.

### Prevalence of *S. japonicum* infection in goats and buffalo from Matang Town

Approximately 60% of the grazing ruminants in Matang Town were buffalo, while the remaining 40% were goats. A total of 6981 fecal samples were examined, i.e., 5714 from buffalo and 1267 from goats. The prevalence of *S. japonicum* infection in buffalo declined nonsignificantly (*P* = 0.33) from 3.62% in 2005 to 2.83% in 2007, after which is remained at a level of about 3.1%. The prevalence in goats was significantly reduced from 13.60% in 2005 to 4.08% in 2010 (*P*<0.01). The prevalence in goats was higher than that in buffalo, with a significant difference in most years (Table 3).

**Table 3:** Prevalence of *S. japonicum* infection in buffalo and goats from Matang Town.

Year	Buffalo			Goat		
	No. examined	No. positive	Prevalence (%)	No. examined	No. positive	Prevalence (%)
2005	967	35	3.62**	206	28	13.60
2006	1089	35	3.21**	280	27	9.46
2007	918	26	2.83*	205	12	5.85#
2008	782	24	3.07*	160	11	6.88#
2009	985	31	3.15	220	9	4.09#
2010	973	30	3.08	196	8	4.08#

*P<0.05;

**P<0.01 comparison of prevalence between buffalo and goats.

# *P*<0.01 comparison of prevalence with that in 2005.

### Bovine prevalence by age in the total study population

Cattle and buffalo were divided into two groups, younger than three years and older than three years, and the prevalence is shown in Table 4. The prevalence in cattle younger than three years was higher than that in cattle over three years, but the difference was not significant. In Nandashan Town, the prevalence in buffalo younger than three years was 3.91–6.99%, which was higher than that found in those older than three years (0.68–4.57%), and there was a significant difference in 2008 and 2010 (*P*<0.01). In Matang Town, the prevalence in buffalo younger than three years was 4.43–6.14%, which was significantly higher than that found in those older than three years (1.10–2.34%), with the exception of 2009 (*P* = 0.14).

**Table 4:** Prevalence (%) of *S. japonicum* infection in bovines of different ages.

Year	younger than three years			older than three years		
	C.N.	B.N.	B.M.	C.N.	B.N.	B.M.
2005	7.55	3.91	6.12**	7.45	1.89	2.34**
2006	4.08	5.32	6.14**	5.41	3.46	1.10**
2007	7.84	6.67	4.43*	6.25	4.57	1.99*
2008	11.9	6.18**	4.92**	3.51	0.68**	1.75**
2009	6.38	5.06	4.48	4.34	2.92	2.65
2010	9.68	6.99**	4.83*	7.14	2.17**	2.34*

*P<0.05;

**P<0.01 comparison of buffaloes younger and older than three years. C.N.,Cattle from Nandashan Town; B.N., Buffaloes from Nandashan Town; B.M., Buffaloes From Matang Town.

### Composition and infection rates in field feces

All positive field feces came from bovines in Nandashan Town. In Matang Town, 61.22% (30) positive field feces were from bovines, while the remaining 38.78% (19) were from goats (Table 5).

**Table 5:** Positive rates in field feces.

Location	Species	April			November/October		
		No. examined	No. positive	Percent positive	No. examined	No. positive	Percent positive
Nandashan	bovine	506	22	4.35	548	22	4.01
Matang	bovine	317	17	5.36	291	13	4.47
	Goat	126	10	7.94	131	9	6.87

The positive rates for field feces in April and November/October are shown in Table 5. The positive rates for bovine feces were 4.35% and 5.36% in April, and 4.01% and 4.47% in November/October, for Nandashan and Matang respectively. The positive rates for goat feces (Matang only) were 7.94% in April and 6.87% in November/October.

## Discussion

A comprehensive control strategy was broadly implemented in China from 2004 to reduce the rate of transmission of *S. japonicum* infection among cattle (and buffalo), humans and snails [11]. The interventions were successful in the majority of the endemic regions, especially the hilly mountainous regions. The country achieved the mid-term goal of reducing the prevalence of schistosomiasis in humans and bovines to <5%, as stated in the document “Outline of mid- and long-term national programme on control and prevention of schistosomiasis (2004–2015)”. Sichuan and Yunnan province reached the national criteria of transmission control, in 2008 and 2009 respectively, by reducing the prevalence in both humans and bovines to less than 1% [6], [12]. The interventions also led to a reduction in human schistosomiasis prevalence in both Nadashang Town and Matang Town. Based on the data from the local human health institute, the prevalence of human schistosomiasis was reduced from 9.8% in 2005 to 3.39% in 2010 in Nadashang Town and from 6.1% to 3.08% in Matang Town (data not shown). The current study found that the number of infected ruminants was reduced significantly from 201 in 2005 to 89 in year 2010. This was due largely to the great decline in the total number of goats present, which was caused by the prohibition of pasturing on some grassy lake beach areas near the residential area and outside but near embankments. However, there was no significant reduction in the prevalence of the infection in ruminants. Data from the local human health institute (not shown in this paper) revealed that there was no reduction in snail prevalence or the density of infected snails in the areas outside embankments in both towns from 2005 to 2010. Thus, the removal of animal-borne *S. japonicum* infections in the Dongting lake region was more difficult compared with other endemic regions of China. This means it is necessary to develop new interventions to reduce the prevalence of infected ruminants in this area.

It has been reported that bovines contribute 80% or more to the local transmission in certain areas [13], [14]. Gray et al. reported that the contribution of *S. japonicum* water buffalo transmission to human infection ranged from 39.1–99.1% and they predicted that the removal of water buffalo transmission would reduce parasite reproductive rates below 1 [15]. In Nandashan Town, all the infected ruminants were bovines and all positive field feces were from bovines. The role of bovines as important nonhuman contributors in the Dongting lake region has received considerable attention in the past three decades, where 90% or more bovines are typically diagnosed and all positive individuals are treated with praziquantel every May/June. In addition, a mass treatment of grazing bovine herds is annually applied in September/October.

In Matang Town, the prevalence of *S. japonicum* infection in goats (4.08–13.60%) was significantly higher than that found in buffalo (2.83–3.62%). We found that 41.8–76.5% of infected ruminants were goat, while 38.78% (19) of 49 positive field feces were also derived from goats. Wang et al. reported that the daily fecal outputs of buffalo and goat were 14667±4671 g and 191±21 g, respectively [13]. We did not count ruminant egg numbers, but we found that the observed miracidial numbers from 10 g goat fecal samples and 50 g buffalo fecal samples were approximately the same (1–4 miracidia) in most infected cases. The highest contribution of goat-transmitted *S. japonicum* to snail infection was 16.47% (in 2005) in Matang Town, if we did not consider *S. japonicum* eggs from infected people and we made rough estimates using the reported formula [13] based on five times the EPG (eggs per gram of feces) from goats versus those from buffalo. This suggests that goats also constitute a major risk factor for schistosomiasis in many areas of the Dongting lake region. Goats were previously not considered as a source of infection. Only cattle and water buffalo are included in the Chinese National Schistosomiasis Control Program. In the Dongting Lake region of Hunan Province, only 20–30% goats were examined annually for *S. japonicum* infection. Positive goats were treated with praziquantel, but the mass treatment applied to grazing ruminants every September/October did not include goats. Thus, we suggest that both pasturing bovines and goats should be treated equally when controlling *S. japonicum* infection in the Dongting lake region.

The high risk areas for domestic ruminants in the Dongting lake region were mainly grassy lake beaches outside embankments where the control of snail populations using chemical molluscicides or habitat modification might be impossible. The majority of cattle and buffalo are used for beef production rather than ploughing, so agricultural mechanization will not significantly reduce the number of grazing animals in future. It would not be possible to completely prohibit of pasturing on grassy lake beaches and to house all domestic ruminants, particularly because pasturing has very little cost. Thus, of the interventions used in recent years, the only effective treatment for infected ruminants and mass treatment were directly targeted at reducing prevalence in ruminants.

Treatment of infected ruminants was usually conducted in early June with a mass treatment in September/October. This was mainly due to pasturing habits, rather than any epidemic regularity of *S. japonicum*. Bovine herds were usually driven back inside embankments every May/June before flooding, and then returned to the areas outside embankments in September after the flooding. The treatment scheme was convenient for veterinary workers, but not necessarily useful for reducing prevalence.

Based on a survey of field feces collected in marshlands, the positive rates for bovine and goat feces were approximately the same in April and October/November. The majority of *S. japonicum* eggs deposited in winter were not the infective source for snails, because of the low temperature. However, the conditions were different after the end of March. From the end of March to early June, *S. japonicum* eggs were constantly excreted by infected ruminants and they became miracidia, which infected the freshwater snail *O. hupensis*. Thus, it is essential to conduct an additional mass treatment in late March or early April in addition to the original treatment scheme.

It was reported that buffaloes are less susceptible to *S. japonicum* infection than cattle [16]. This was confirmed in the present study in Nandashan Town, where the prevalence in cattle (4.40–8.47%) was higher than that in buffalo (2.77–5.59%) in each study year, with significant differences in 2005 and 2008. The present study also found that the prevalence in goats was significantly higher than that in buffalo in most study years, while buffalo younger than three years were more susceptible than those over three years. However, there was no similar significant age difference in cattle. Those data enrich our knowledge of the host-parasite- relationship in *S. japonicum*.
